# Web-Based Self-Help for Problematic Alcohol Use: a Large Naturalistic Study

**DOI:** 10.1007/s12529-016-9618-z

**Published:** 2016-11-29

**Authors:** Magnus Johansson, Kristina Sinadinovic, Anders Hammarberg, Christopher Sundström, Ulric Hermansson, Sven Andreasson, Anne H Berman

**Affiliations:** 1 Department of Public Health, Karolinska Institutet, Stockholm, Sweden; 2 Department of Clinical Neuroscience, Centre for Psychiatry Research, Karolinska Institutet, Stockholm, Sweden; 3 Stockholm Center for Dependency Disorders, Stockholm, Sweden

**Keywords:** Alcohol, Harmful drinking, Substance use disorders, Internet, Cognitive behavioral, Treatment program, eHealth

## Abstract

**Purpose:**

This observational study examined user characteristics, intervention use patterns, and variables associated with reductions in alcohol consumption for anonymous Internet help-seekers using a Web-based self-help program.

**Method:**

A Web-based cognitive behavioral therapy (CBT) program with eight modules delivered over 10 weeks was offered to participants with at least hazardous use of alcohol according to the Alcohol Use Disorders Identification Test (AUDIT) (*n* = 4165). At baseline and 10-week follow-up, participants completed the Timeline-followback (TLFB), AUDIT, Drug Use Disorders Identification Test (DUDIT), Hospital Anxiety and Depression Scale (HADS), EuroQol-5 dimension (EQ-5D), World Health Organization Quality of Life Scale-abbreviated version (WHOQOL-BREF), Readiness to Change Questionnaire (RCQ), and Readiness Ruler. Follow-up completers and non-completers were compared at baseline, and follow-up completer outcomes were reported. Predictors of change in drinking behavior were evaluated at follow-up.

**Results:**

Registered users were 41.88 years old on average (SD = 12.36), and 52 % were women; the mean baseline number of drinks during the past week was 27.27 (SD = 17.92) with 62 % in the AUDIT category of probable dependence and only 7 % having low-risk consumption according to public health guidelines. At follow-up (*n* = 1043), 53 % showed a clinically significant change to a lower level of alcohol use (χ^2^ = 254.403, *p* < 0.001); the mean alcohol consumption fell (*t* = 22.841, *p* < 0.001) and the proportion with low-risk consumption rose to 40 %. Being male, scoring higher on baseline readiness, completing the program, and accessing other support predicted low-risk drinking and clinically significant change to a lower level of alcohol use at follow-up.

**Conclusion:**

A publicly available Web-based program for managing problematic alcohol use attracted users with considerable alcohol- and health-related problems, which were changed to lower severity for follow-up completers.

## Introduction

Excessive consumption of alcohol is one of the leading risk factors causing a great proportion of disability-adjusted life years and death in the world [1]. In Sweden, alcohol consumption has increased during the last 20 years, although recent years have shown a decline in consumption [2]. In 2014, 15 % of men and 12 % of women in Sweden had a risky consumption of alcohol [3]; about 4 % of individuals in the Swedish adult population meet criteria for alcohol dependence [4]. However, only about 26,000 people in Sweden were estimated to have received specialized care within the health-care system for an alcohol-related diagnosis in 2008 [5]. Only a minority of individuals with alcohol dependence seek treatment. Common reasons for not seeking help are fear of stigmatization, shame, and the will to resolve the problem without external help [6, 7]. Both Swedish and international experiences show that Web-based interventions may reach those who have problematic alcohol use [8, 9], as well as those who to a lesser extent come into contact with addiction services [10]. Sweden is one of the countries in the world where Internet use is highest and most widespread in the community. In the Swedish population, 91 % are Internet users [11]. Individuals with alcohol dependence who participated in a Swedish focus-group and interview study indicated Internet as an attractive first step for assessment of alcohol use and guidance to treatment but not for actual treatment [12].

The research field of Web-based interventions for reducing problematic alcohol use can be considered quite new, and researchers are still struggling with methodological problems [13, 14]. Published studies have shown small effects in terms of reduced alcohol consumption but have often been limited to student populations or single session interventions [15, 16]. Extended Web-based interventions are often based on principles of cognitive behavioral therapy (CBT) and motivational interviewing [17] and are intended to be used continuously for a number of weeks. Compared to single-session interventions, effect sizes for more extended interventions seem to be somewhat larger (Hedges’ g = 0.61 compared to 0.27) [18].

We have identified three prior naturalistic studies on extended Web-based interventions showing that users had a significant decrease in dependence symptoms [19] or reduced their drinking in ways that correspond with participants in randomized controlled trials, with about 18 % within guidelines for low risk at follow-up [20, 21]. However, much is unknown about members of the general public that seek out and participate in Web-based interventions on their own or how these interventions are used [9]. There is little evidence for long-term or clinically significant effects, such as meeting drinking limits or reducing binge drinking. The current research literature shows a lack of information on possible dose-response relationships or the possible importance of complementary care. Results regarding which populations are more or less likely to benefit are inconclusive [13].

The purposes of this observational study were to examine user characteristics and patterns of intervention use for a Web-based self-help program offered to Internet help seekers and to examine which factors were associated with reductions in alcohol consumption. The specific research questions were as follows:

- What are the characteristics of individuals who sign up for a Web-based self-help program for managing problematic alcohol use, which is freely offered to the public within a research study, in terms of self-rated levels of alcohol and drug use, alcohol-related problems, health problems, quality of life, and readiness to change?
- What patterns of program use, working alliance ratings, and levels of access to other support occur among individuals registered for a Web-based self-help program for managing problematic alcohol use?
- How do baseline factors such as severity of alcohol use, symptoms of anxiety and depression, quality of life, health, readiness to change, working alliance, and the level of program usage influence self-reported alcohol consumption outcomes among individuals registered for a Web-based self-help program for managing problematic alcohol use?

## Method

### Study Design

This study used a pre-post observational design where all included participants received access to the intervention.

### Participants and Recruitment

Participants were recruited online through the Swedish Internet site alkoholhjalpen.se, an open access Web site that provides information and a discussion forum to individuals seeking help for their alcohol consumption. The site was owned by the Swedish Public Health Agency until 2015 when it was transferred to the Stockholm Center for Dependency Disorders. The site has been publicly accessible since 2007 and had approximately 5000 visitors every month during 2013 and 2014.

### Procedure

During a period of 2 years, between January 4, 2013 and January 3, 2015, a brief informational text about a Web-based program for problematic alcohol use was available at the top of the front page of alkoholhjalpen.se. Those interested who clicked on the link were directed to an information page with an overview of the study and information about the handling of personal data. Individuals who wanted to participate were instructed to give informed consent by clicking a “yes” button at the bottom of the Web page. They were then directed to a screening page where they were required to indicate their gender and age and fill in Web-based Swedish versions of the Alcohol Use Identification Test (AUDIT [22]) and the first question of the Drug Use Disorders Identification Test (DUDIT [23]); registrants who scored >0 filled in all 11 items. Those with an AUDIT score indicating at least hazardous use, i.e., a score of ≥6 for women and ≥8 for men, and who were at least 18 years of age were given a brief message informing them of their problematic alcohol use and offered participation in the study with access to the program. In order to gain access to the intervention, participants had to create a personal account with a username and password for secure access to the intervention Web site eChange, as well as contact data in the form of an e-mail address and phone number, to be used only to send out reminders. After the personal account was created, the participant was asked to fill out baseline measures (see below). Registrants without problematic alcohol use were informed of their non-problematic use and that they therefore did not qualify for the study.

### The Web-Based Self-Help Program, eChange

Following completion of the baseline questionnaires, all participants were given access to the Web-based self-help program for managing problematic alcohol use eChange. No counselor guidance was available. The program was delivered via the open-source platform Drupal (drupal.org) programmed by the first author. Communication between the program server and the user was encrypted and protected with an individual login name and a password. The program was a Swedish translation and adaptation of the Dutch program Therapy Alcohol Online [24] which, in turn, is based on a Dutch adaptation of the CBT and MI manuals from project MATCH [25, 26]. The program consists of eight modules with 2–3 pages of reading material per module along with exercises where the participant can write answers in free text or choose from pre-formulated options. The following modules are included in the program: (1) analyzing advantages and disadvantages of drinking (decisional balance) and exploring motivation to change; (2) setting an alcohol consumption goal (moderation or abstaining from drinking); (3) learning self-control skills; (4) identifying risk situations; (5) managing craving; (6) handling emotions; (7) dealing with social pressure; and (8) developing a crisis plan.

During the first 15 months of the study, the first four modules were released to the user consecutively, *once a week*; modules 5–7 were simultaneously released during the fifth week for use during weeks 5 and 6, and module 8 was released at week 7. Then, there was a 3-week gap between weeks 7 and 10 to give the participant an opportunity to try out the techniques taught in the program. Each participant received an e-mail every time a new module was made accessible. We observed that module completion rates were low and made an attempt to improve completion rates by introducing a change on March 31, 2014, where *all modules were released at once* to the users directly after baseline measurement. This meant that users could access any of the modules at any time. They were, nonetheless, given a recommendation to follow the sequence described above, and they were still sent e-mail reminders and a new start message at the beginning of weeks 1–5 and 7, with recommendations on which module to work on. During the treatment period, all participants were encouraged to register craving as well as daily alcohol consumption in a calendar included in the program. Users could access continual feedback about their progress through the calendar’s statistics page, where they could see their average personal consumption as well as the number of days drinking, the number of days sober, and binge drinking occasions. In addition, they could also view a personal summary of their own risk situations with information on where they drank and the level of craving they had experienced on each risk situation occasion. An electronic private diary was also available for the participants. All participants could access the self-help program whenever they wanted for an unlimited number of times during the study as well as up to approximately 9 months after the last follow-up, until December 1, 2015. Users were encouraged to use as much of the program as they wanted.

### Primary Outcomes

*Low-risk consumption* was defined as alcohol consumption within Swedish guidelines of no more than 9 drinks for women and no more than 14 drinks for men during the past week and no more than 3 drinks for women and 4 drinks for men (binge drinking) on any one of the days during the past week. One drink is equal to 12 g of alcohol according to the Swedish definition.

A *clinically significant change in the level of alcohol use* defined as moving from one alcohol use category in AUDIT at baseline to another category at follow-up. Each individual was assigned to an alcohol use level category based on their total AUDIT score. A score of ≥20 indicates “probable dependence,” a score of 16–19 indicates “harmful use,” and scores of 8–15 for men and 6–15 for women indicate hazardous use [27].

### Follow-up

Ten weeks post-registration (3 weeks after completion of last program module), participants were e-mailed and asked to log into the system to complete the follow-up questionnaires consisting of the same questionnaires as at baseline. Participants who did not respond to this initial request received two automated e-mail reminders followed by one manual e-mail reminder from the first author and a mobile text message. Due to high dropout levels in the early phase of the study, the procedure was revised from March 31, 2014 and onwards, by adding up to 10 additional automated e-mail reminders during a 2-week period after the 10-week follow-up time point. Once the participant filled in the follow-up measures, no further reminders were sent.

### Instruments

*The Timeline Follow Back* (*TLFB* [28]) was used to record the number of standard drinks of alcohol consumed each day during the past 7 days, to assess low- or high-risk consumption categories as well as changes in the alcohol consumption. TLFB administered via computer has been found to yield data that correlate with paper and pencil administration [29].

*AUDIT* is a well-established and widely used 10-item instrument for measuring *alcohol use*, including alcohol consumption as and signs of harm or dependence related to alcohol consumption [30]. The Internet version has shown Cronbach’s α values of 0.80–0.93 [31], and the Swedish paper version has yielded Cronbach’s α values of 0.81–0.82 [22].

*DUDIT* is an 11-item questionnaire designed to assess patterns of *drug consumption and drug-related problems* [23, 31]. The first item was used as an indicator of whether or not the individual had any drug problems; only positive responses on the first question led to full completion of the questionnaire.

*The Hospital Anxiety and Depression Scale* (*HADS*) comprises 14 items on two subscales to measure *anxiety and depression* symptoms [32, 33].

*The World Health Organization Quality of Life Scale-abbreviated version* (*WHOQOL-BREF*) consists of 26 items and measures quality of life on four domains: physical, psychological, social, and environmental [34].

*EuroQol-5 dimension* (*EQ-5D*) assesses health-related *quality of life* and consists of five items covering the dimensions of mobility, self-care, usual activity, pain/discomfort, and anxiety/discomfort, scored on a 1 to 5 Likert scale. From the 5 items, an index score was calculated with Crosswalk value sets, using the United Kingdom as a reference [35]. The EQ-5D also includes a VAS between 0 and 100 regarding the respondent’s current health status [36].

*The Readiness to Change Questionnaire* (*RCQ*) assesses the respondent’s *motivation* for change with 12 questions covering the pre-contemplation, contemplation, and action dimensions of the Trans-Theoretical model of change [37, 38].

*The Readiness Ruler* consists of a Visual Analog Scale (VAS), where users give their responses on a scale of 0–10 in relation to the statements “I am not ready to change my drinking habits” (0) and “I am very much ready to change my drinking habits” (10). The scale has been shown to predict future alcohol consumption [39].

Additional questionnaires at follow-up were (a) evaluation of Web-based treatment originally developed by a Dutch research team [40] and (b) questions concerning involvement in other support (e.g., treatment or informal) for problematic alcohol use during the treatment period [41].

The program use variable as used in this study describes number of completed module exercises. Participants who completed any 5 out of 7 possible modules were considered to be *program completers.* Use of calendar registration of alcohol consumption and use of private diary was defined as having used them more than once during the program.

After completing each module in the program, participants were encouraged to rate the working alliance with the program via the *Session rating scale* (SRS) [42]. The SRS consists of four VAS scales corresponding to Bordin’s definition of the therapeutic alliance [43]. For the purposes of this study, the SRS was adapted for use with a Web-based program.

### Statistical Analysis

No power analysis was calculated prior to the initiation of this study due to its observational character. Descriptive statistics were used to describe baseline characteristics. Chi-square and *t* tests were used to assess whether there were baseline differences between completers and non-completers and between baseline and follow-up. All the analyses were done on complete cases for each measure. In order to evaluate predictors of changed drinking behavior at follow-up, two multiple logistic regressions were conducted. The dependent dichotomous variables were (a) low- or high-risk drinking based on the TLFB and (b) clinical change to lower categories of alcohol use based on AUDIT (yes/no). Potential predictor variables were age, gender, number of drinks, drinking days, and binge-drinking days the week before baseline, alcohol use (AUDIT), drug use, health-index and self-rated health today (EQ-5D), the physical, psychological, social, and environmental domains from WHOQOL-BREF, readiness ruler and action score (RCQ) at baseline, as well as completion of program, use of calendar or electronic diary, having talked to someone, had contact with professional or used pharmacological treatment to change alcohol consumption. Non-significant variables were removed backwards from the model, one by one, until −2 log likelihood deteriorated significantly. Goodness of fit of the model was determined by the Hosmer­Lemeshow test. All statistical tests were two-sided, with *p* ≤ 0.05 considered as significant. All analyses were performed using IBM SPSS Statistics for MacOS X, Version 23 (IBM Corp, Armonk, NY, USA).

**Fig. 1:**
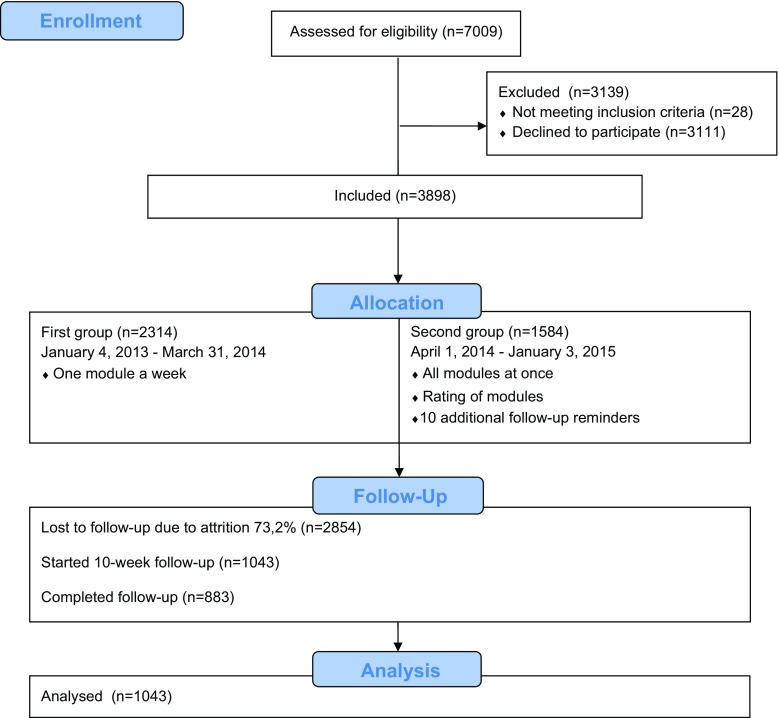
Flow diagram

The number of screenings completed and users registered between January 4, 2013 and January 3, 2015 are shown in Fig. 1. The most frequent days for screenings (38 %) were Sundays and Mondays. The number of screenings completed per month (M = 280.36; SD = 79.82) and the number of monthly accounts created (M = 155.92; SD = 56.76) remained stable during the 2 years when participants were recruited to the study.

### Ethics and Trial Registration

The study was approved by the Stockholm Regional Ethical Review Board (ref nr 2013/686–31/5, May 2, 2013), and the trial was registered at Clinicaltrials.gov (NCT02283593). Informed consent was obtained from all individual participants included in the study.

## Results

### Descriptive Characteristics at Baseline

Of the registered users, over half were women and the mean age was high. The users’ mean number of drinks and binge drinking days during the week before baseline was well over recommended guidelines for low-risk drinking and a majority had levels of alcohol use corresponding to alcohol dependence. A large proportion of the users reported problems with depression or anxiety. Baseline characteristics for all individuals included in the study and differences between those who participated in follow-up and those who were lost to follow-up are shown in Table 1.

**Table 1:** Baseline characteristics for study participants, with comparisons between those lost to follow-up and those who completed follow-up, as well as 10-week (post-treatment) follow-up, with comparisons between baseline and follow-up results

	At baseline									At follow-up					
	All participants		Lost to follow-up		Participated in follow-up		Statistical parameter		*p*			Statistical parameter		*p*	Cohen’s *d*
	(*n* = 3897)		(*n* = 2854)		(*n* = 1043)										
Age, years, M (SD)	41.88	(12.36)	40.68	(12.31)	45.18	(11.90)	t =	−10.177	<0.001						
% Gender, male/female	48/52		48/52		50/50		χ^2^ =	0.965	0.326						
AUDIT (*n* = 3897)										(*n* = 1012)					
AUDIT score	21.39	(6.08)	21.82	(6.12)	20.24	(5.81)	t =	7.414	<0.001	14.84	(6.47)	t =	29.831	<0.001	0.98
Alcohol use categories (% in non-problematic/hazardous/harmful/probable dependence)	0/18/20/62		0/17/18/65		0/22/24/54		χ^2^ =	39.122	<0.001	9/48/21/22		χ^2^ =	254.403	<0.001	
Drug use (% DUDIT >0)	13		14		10					8					
TLFB (*n* = 3867)										(*n* = 968)					
Drinks past week	27.27	(17.92)	28.10	(18.66)	25.00	(15.52)	t =	4.777	<0.001	12.96	(13.79)	t =	22.841	<0.001	0.74
No of drinking days/week	4.36	(2.02)	4.35	(2.03)	4.37	(1.99)	t =	−0.274	0.784	2.93	(2.19)	t =	22.805	<0.001	0.66
No of binge drinking days	3.03	(2.00)	3.10	(2.02)	2.84	(1.94)	t =	3.571	<0.001	1.37	(1.71)	t =	20.476	<0.001	0.74
Low-risk drinking (% ≤ 9/14 drinks/week for women/men)	5		5		7		χ^2^ =	3.904	0.048	40		χ^2^ =	42.988	<0.001	
HADS (*n* = 3897)										(*n* = 896)					
Anxiety	6.53	(4.02)	6.74	(4.08)	5.97	(3.77)	t =	5.291	<0.001	7.03	(4.52)	t =	17.801	<0.001	0.70
Clinical status (% ≥11)	73.1		69.1		74.6		χ^2^ =	11.589	0.001	16		χ^2^ =	206.360		
Depression	10.51	(4.70)	10.77	(4.74)	9.82	(4.53)	t =	5.673	<0.001	3.86	(3.50)	t =	20.966	<0.001	0.60
Clinical status (% ≥11)	22		17		24		χ^2^ =	21.582	<0.001	4		χ^2^ =	164.689	<0.001	
WHOQOL-Bref (*n* = 3897)										(*n* = 891)					
PHYS	12.7	(2.51)	12.58	(2.53)	13.02	(2.40)	t =	−4.810	<0.001	14.40	(2.58)	t =	−18.372	<0.001	−0.62
PSYCH	11.19	(2.79)	11.05	(2.84)	11.59	(2.62)	t =	−5.345	<0.001	13.18	(2.77)	t =	−20.131	<0.001	−0.68
SOCIAL	11.93	(3.11)	11.83	(3.17)	12.20	(2.91)	t =	−3.231	0.001	13.46	(3.02)	t =	−12.809	<0.001	−0.43
ENVIR	14.67	(2.49)	14.49	(2.53)	15.18	(2.29)	t =	−7.769	<0.001	15.75	(2.27)	t =	−8.904		−0.30
EQ5D (*n* = 3897)										(*n* = 890)					
EQ5D index	0.70	(0.20)	0.70	(0.20)	0.73	(0.17)	t =	−0.327	0.755	0.78	(0.18)	t =	−16.636	<0.001	−0.34
EQ5D Health today	58.32	(22.25)	57.22	(22.65)	61.34	(20.83)	t =	−5.132	<0.001	73.07	(18.66)	t =	−10.099	<0.001	−0.56
RTCQ (*n* = 3897)										*n* = 883					
Action score	0.46	(4.04)	0.28	(4.05)	0.96	(3.97)	t =	−4.654	<0.001	4.92	(3.43)	t =	−26.150	<0.001	−0.88
RTCQ categories(% in Pre-contemplation/Contemplation/Action)	0/91/8		1/92/8		0/90/10		χ^2^ =	10.723	0.005	1/41/58		χ^2^ =	21.667	<0.001	
Readiness ruler	8.37	(1.89)	8.34	(1.94)	8.46	(1.75)	t =	−1.810	0.07	7.99	(2.17)	t =	6.521	0.001	0.22

### Program Use

Out of 7 module exercises, the mean number completed was 2.2 (SD = 2.2). The greatest loss of users was in the early phase of program participation, with 34 % of all participants completing the first three modules; 44 % used the calendar more than once and 35 % used the private diary on more than one occasion. A significantly smaller proportion (13 vs 16 %, χ^2^ = 11.863, *p* = 0.001) completed the program among the participants who were given access to all modules at once compared to the initial participants who were allocated one module per week.

### Attrition

Attrition was defined as not participating in the follow-up. The attrition rate was 73 % with 1043 users participating in follow-up. There were no differences in attrition between the users recruited before March 31, 2014 and those recruited afterwards, who received all modules from start and 10 more reminders to complete the follow-up. Users completing the follow-up showed a younger mean age, lower baseline levels of alcohol consumption, alcohol use, as well as anxiety and depression scores. Users completing the follow-up also had higher quality of life in all dimensions but slightly more health problems than those lost to follow-up. Follow-up group participants were not more motivated to change their drinking according to their mean score on the readiness ruler, but a slightly higher percentage were in the action stage and 44 % completed the program.

### Alliance

Measuring alliance with the service during the program was not feasible as attempted in this study. Only 1277 measures were completed by 720 participants (18.5 %, M = 29.2, SD = 8.7), where 306 completed two or more measures. A decrease in alliance score between measures (*n* = 140, 46%) was not associated with program completion or achieving low-risk drinking at follow-up.

### Involvement in Other Support

Out of the participants that completed the follow-up, 61 % had talked to someone about their alcohol habits during the time when they used the program, including 56 % who talked to a family member or friend and 20 % that had contact with a professional. Pharmacological treatment had been used by 6 %, and 13 % had used other Internet resources.

### Outcomes at Follow-up

Among participants in the follow-up group, 40 % reported low-risk consumption (TLFB) at follow-up, compared to 7 % at baseline. Between baseline and follow-up, 53 % of the participants made a clinically significant change to a lower level of alcohol use, according to AUDIT. The within-group effect size (Cohen’s *d*) for number of drinks consumed in the past week (TLFB) was 0.74; the effect size for change in alcohol use (AUDIT-score) was *d* = 0.98. The change in alcohol use varied depending on which category of alcohol use that the participants belonged to at baseline. Participants with harmful use (*d* = 1.25) and probable dependence (*d* = 1.21) had larger within-group effects than participants with hazardous use (*d* = 0.72).

In readiness to change, 55 % moved from the contemplation stage at baseline to the action stage at follow-up. Significant changes in alcohol and several other measures occurred between baseline and follow-up. Table 1 shows follow-up scores on all the outcome measures.

Among follow-up participants, 24 % (*n* = 249) changed their alcohol use to a more severe category according to AUDIT, or stayed within the highest category corresponding to probable dependence. In addition, 3 % (*n* = 23) moved to a lower category of readiness to change their alcohol habits, 19 % (*n* = 174) moved from no problems with anxiety to mild or clinical problems or continued to have clinical problems, and 7 % (*n* = 64) moved from no problems with depression to mild or clinical problems or showed continued clinical problems between baseline and follow-up according to HADS categories.

### Predictors of Drinking Outcome

The regression model with low-risk consumption at follow-up (TLFB) as dependent variable showed a statistically significant independent contribution of ten of the potential predictor variables. Women were less likely (OR = 0.63) to have low-risk consumption at follow-up compared to men. Receiving all modules at once (OR = 1.46), completing the program (OR = 1.47), having spoken with someone about their drinking since registering for the study (OR = 1.32), and having a higher score on the readiness ruler (OR = 1.15) were also associated with an increased likelihood of low-risk consumption at follow-up. Participants who had used the private diary (OR = 0.70) had lower odds of low-risk consumption. Individuals with more drinking days (OR = 0.90) and more binge-drinking days (OR = 0.78) or those who used other drugs (OR = 0.90) at baseline were less likely to have low-risk consumption at follow-up, whereas people with higher score of depression (OR = 1.04) were slightly more likely to have this outcome.

The regression model developed to predict clinically significant change to a lower level of alcohol use at follow-up (AUDIT) showed a significant contribution of seven predictor variables. Women (OR = 0.55), users who had contact with a professional about their drinking (OR = 0.68) or used pharmacological treatment since registering (OR = 0.66), were less likely to show a clinically significant change to a lower level of alcohol use. Receiving all modules at once (OR = 1.60) as well as increased scores on the readiness ruler (OR = 1.14) were factors associated with a higher likelihood of clinically significant change to a lower level of alcohol use. Having a higher number of drinks during the last week was associated with slightly lower odds (OR = 0.98), but a higher AUDIT score at baseline was associated with slightly higher odds (OR = 1.06) of clinically significant change to a lower level of alcohol use. Both regression models are shown in Table 2.

**Table 2:** Two logical regression models developed to predict drinking outcome at follow-up (*n* = 861)

Dependent variable	Low risk drinking at follow-up				Clinically significant reduction in alcohol problems ^a^			
Predictor variables	B	S.E.	*p*	OR	B	S.E.	*p*	OR
Woman	−0.46	0.15	0.002	0.63	−0.41	0.15	0.005	0.66
Drinks last week				-	−0.02	0.01	0.001	0.98
Days drinking	−0.11	0.04	0.015	0.90				-
Days binge drinking	−0.25	0.05	<0.001	0.78				-
AUDIT score				-	0.05	0.02	<0.001	1.06
Drugs	−0.50	0.26	0.05	0.61				-
Depression	0.04	0.02	0.045	1.04				-
Readiness scale VAS	0.14	0.05	0.003	1.15	0.13	0.04	0.002	1.14
Completed program	0.39	0.16	0.018	1.47				-
User diary	−0.35	0.17	0.036	0.70				-
All modules at once	0.38	0.16	0.015	1.46	0.47	0.15	0.001	1.60
Spoke to someone	0.28	0.16	0.076	1.32				-
Contact with profess.				-	−0.39	0.19	0.037	0.68
Pharmacological treatm.				-	−0.61	0.32	0.058	0.55
Constant	−0.79	0.48	0.101	0.45	−1.49	0.44	0.001	0.23
Nagelkerke *R* ^2^	0.16				0.08			
Percent correct	65.7				60.2			
Hosmer and Lemeshow test χ2 (p)	12.42 (0.134)				6.05 (0.641)			

Predictors that did not contribute significantly to and where excluded from both models: age, anxiety, health index and VAS (EQ-5D), the physical, psychological, social and environmental quality of life (WHOQOL-BREF), action score (RCQ), and having used calendar

^a^A shift from a more problematic category (hazardous/harmful/probable dependence) to a less problematic category at least one level lower (no problematic use/hazardous/harmful)

*OR* odds ratio

“-” indicates that predictor was excluded because it did not contribute significantly to the model

## Discussion

The purpose of the present study was to investigate the usage of a publicly available Swedish Web-based program for managing problematic alcohol use, with a focus on characteristics of program users, patterns of program use, and the relation between user characteristics, program use, and drinking outcomes.

The main findings are that users were older compared to previous Web-based studies and the gender balance was about equal. Users had a high number of drinks consumed in the past week at baseline, had alcohol use corresponding to alcohol dependence, clinical levels of anxiety or depression symptoms, high levels of health-related problems, and low quality of life at baseline. Users indicated a high average readiness to change their alcohol consumption at baseline. Most of the users only used a small proportion of the program components. A majority of users were lost to follow-up, completers had lower alcohol consumption, alcohol use, level of anxiety, depression, and showed higher scores on quality of life and health at follow-up compared to baseline. Study completers who were male rather than women, who were more ready to change at baseline, and who completed the program or talked to someone about their drinking habits were more likely to have low-risk consumption or clinically significant change to a lower category of alcohol use at follow-up.

The participants in the present study showed a higher mean age (41.88 years) than participants in previous studies of Web-based interventions aimed at the general public [9, 15], but approximately the same age as an earlier Swedish study targeting individuals seeking help for problematic alcohol use over the Internet [41]. In line with previous studies of Web-based interventions in Sweden and elsewhere, the proportions of men and women were approximately equal [8, 13, 41]. This is in contrast to regular Swedish alcohol dependence care, where a majority of users are men [5].

The users had problems in several areas. The mean number of drinks consumed by the participants was equal to individuals in a Swedish study of brief treatment for problematic alcohol use in a clinical setting [44]. Most users had alcohol use (AUDIT) corresponding to probable dependence, and many of them had symptoms of depression or anxiety above the clinical cut-off that would warrant further assessment and additional treatment. The users had more problems with their health compared with participants in a large controlled study of a similar British Web-based intervention [45]. The participants’ scores on different dimensions of quality of life were low compared to a sample from the general population [46] in Norway, a country very similar to Sweden.

Concerning patterns of program use, several important conclusions may be drawn from the present study. The common mode of delivering Web-based programs has been to deliver one module at a time. In the present study, this mode of program delivery had a positive correlation with completion of the program. Contrary to what might have been expected from this result, individuals who were given simultaneous access to all modules at the start of the program showed higher odds of low-risk drinking and clinically significant change in alcohol use compared to participants who received modules one at a time. One explanation might be that since most users visited the intervention site on only one or two occasions, they might have been able to get a higher dose of the interventions in fewer visits.

Study completers showed a significant change in most outcome variables. There was a larger change in AUDIT scores for participants who were followed up in the present study, compared to changes observed after 12 months in a population sample for individuals in each category of alcohol use; hazardous: *d* = 0.72 vs 0.26, harmful: *d* = 1.24 vs 0.59, and probable dependence: *d* = 1.21 vs 0.57 [47]. This suggests that the change in alcohol use in the present study is larger than what could be explained by effects of assessment reactivity [48] and regression to the mean [49]. However, the fact that participants in this study were help seekers and that larger attrition occurred among those who did not change their drinking could explain the observed change. A randomized controlled study of the intervention is needed to clarify if it has any effects.

A number of users indicated worse alcohol-related problems at follow-up compared to baseline. Also, participants who were lost to follow-up showed higher levels of alcohol consumption and problems related to alcohol and health at baseline. The present study indicates that more focus is warranted on methods to treat users in more need of support for, e.g., psychiatric or somatic problems to prevent possible deterioration.

Logistic regression analysis showed that women had a lower chance of achieving low-risk drinking or clinically significant changed alcohol use. These results differ from those obtained in a Dutch study [50] where being female predicted a better treatment response for those receiving Web-based intervention. Similar to the results in an earlier Swedish study [41], talking to someone (family, friend, or professional) about alcohol habits during the intervention led to higher odds of achieving low-risk drinking. A recently published pilot study showed larger effects for a guided version compared to a self-help version of the same intervention used in the present study [51]. It is proposed that future Web-based interventions include more precise instructions about the additional value of other support beside the Web-based program.

Readiness to change has been shown to be an important factor moderating the outcome of Web-based programs [39, 52]. In the current study, the readiness ruler score at baseline predicted both achieving low-risk drinking at follow-up and clinically significant changes in level of alcohol use. In addition, a large number of participants indicated a transition from the contemplation stage at baseline to action stage at the end of program, according to the RCQ. The most common day of registering for the study was Mondays and Sundays. This probably reflects that the most common time for alcohol consumption in Sweden is during weekends, and that people are more apt to seek help when they experience negative consequences of their drinking.

Using the private electronic diary lowered the probability for low-risk alcohol consumption and change in alcohol use at follow-up. This might be explained by the finding that what users write about alcohol use can modulate motivation and predict change [53]. If users mainly write about reasons for alcohol use, there is a risk that writing might help maintain continued drinking. Another explanation might be that that frequent use of the private diary in itself is a proxy indicator of more severe alcohol problems, and hence reflects a selection bias when interpreting the results.

### Strengths and Limitations

This is the largest study of Web-based interventions for alcohol in the Swedish general public to date. Participation in this study was accessible to all help-seeking individuals with at least hazardous use that visited a well-established Swedish Web portal. The service was the only Web-based extended program for reducing alcohol consumption that was offered to the public in Sweden during the study period. The study provides knowledge about individuals that use these kinds of interventions when offered publicly. Because of the high degree of Internet usage in Sweden, the results can provide a better understanding of users beyond early adopters of Web-based interventions. The large number of participants gave sufficient power to investigate how different factors like use of program components and complementary care might affect alcohol consumption. The study also provided new information on which populations are more likely to benefit from Web-based interventions for alcohol.

Web-based interventions have had problems with attrition [54]. The attrition was even higher in this naturalistic setting than in previous studies. Most users only completed one or two modules, and only one out of four chose to participate in the follow-up. The attrition might have been affected by the large number of questions in the follow-up. Conclusions cannot be drawn regarding all users of these kinds of programs, and only limited conclusions can be drawn about how program usage might affect outcome.

To understand more about how factors like gender modulate outcomes in Web-based programs, there is a need for large studies with better retention. One challenge in all Web-based studies is to retain larger proportions of the participants without selecting only those that agree to be identified. Better tailoring of interventions and study designs to user needs should be tried out, e.g., by identifying and addressing co-occurring problems or helping people transition between Web-based and other types of support. Other possibilities for future studies could be to investigate in greater depth how initial readiness for change might be supported by Web-based interventions or to analyze users’ electronic journal entries and how they might relate to drinking outcomes in Web-based interventions.

## Conclusions

A Web-based program for managing problematic alcohol use attracted users with considerable problems with alcohol and health, which showed changes at the end of the program for users that participated in follow-up. This study has provided findings that can help in the development of future Web-based interventions and in designing future studies.
